# *dtorsin*, the *Drosophila* Ortholog of the Early-Onset Dystonia *TOR1A* (*DYT1*), Plays a Novel Role in Dopamine Metabolism

**DOI:** 10.1371/journal.pone.0026183

**Published:** 2011-10-12

**Authors:** Noriko Wakabayashi-Ito, Olugbenga M. Doherty, Hideaki Moriyama, Xandra O. Breakefield, James F. Gusella, Janis M. O'Donnell, Naoto Ito

**Affiliations:** 1 Center for Human Genetic Research, Massachusetts General Hospital, Boston, Massachusetts, United States of America; 2 Department of Biological Sciences, University of Alabama, Tuscaloosa, Alabama, United States of America; 3 School of Biological Science, University of Nebraska-Lincoln, Lincoln, Nebraska, United States of America; 4 Department of Neurology and Radiology, Massachusetts General Hospital and Program in Neuroscience, Harvard Medical School, Boston, Massachusetts, United States of America; Columbia University, United States of America

## Abstract

Dystonia represents the third most common movement disorder in humans. At least 15 genetic loci (DYT1-15) have been identified and some of these genes have been cloned. *TOR1A* (formally *DYT1*), the gene responsible for the most common primary hereditary dystonia, encodes torsinA, an AAA ATPase family protein. However, the function of torsinA has yet to be fully understood. Here, we have generated and characterized a complete loss-of-function mutant for *dtorsin*, the only *Drosophila* ortholog of *TOR1A*. Null mutation of the X-linked *dtorsin* was semi-lethal with most male flies dying by the pre-pupal stage and the few surviving adults being sterile and slow moving, with reduced cuticle pigmentation and thin, short bristles. Third instar male larvae exhibited locomotion defects that were rescued by feeding dopamine. Moreover, biochemical analysis revealed that the brains of third instar larvae and adults heterozygous for the loss-of-function *dtorsin* mutation had significantly reduced dopamine levels. The *dtorsin* mutant showed a very strong genetic interaction with *Pu* (*Punch*: GTP cyclohydrolase), the ortholog of the human gene underlying DYT14 dystonia. Biochemical analyses revealed a severe reduction of GTP cyclohydrolase protein and activity, suggesting that *dtorsin* plays a novel role in dopamine metabolism as a positive-regulator of GTP cyclohydrolase protein. This *dtorsin* mutant line will be valuable for understanding this relationship and potentially other novel torsin functions that could play a role in human dystonia.

## Introduction

Dystonia is a movement disorder characterized by sustained muscle contraction. While 15 different gene loci have been implicated in primary hereditary dystonia (DYT1-DYT15) [1], the most common and severe form, early-onset dystonia (also known as DYT1 dystonia) is due to mutation in *TOR1A* (formerly *DYT1*) and displays dominant inheritance with reduced penetrance [2]. *TOR1A* encodes torsinA, a 332 amino acid protein from the AAA ATPase family. TorsinA is localized in the lumen of the endoplasmic reticulum and the nuclear envelope [2], but its function is not fully understood. Most cases of DYT1 dystonia are caused by a single mutation, a 3 bp (GAG) deletion that results in the loss of a glutamic acid residue in the carboxyl terminal region of torsinA [3].

In mammals, four paralogous torsin genes (*TOR1A*, *TOR1B*, *TOR2A*, and *TOR3A*) have been described, while *Drosophila melanogaster* has only a single torsin gene, *dtorsin (*previously known as *torp4a*: CG3024) in 4C11 on the X chromosome [4]. The *dtorsin*-encoded protein, Dtorsin, comprises 339 amino acids with 31.9% identity to human torsinA and also displays the characteristic features of the AAA ATPase gene family [5]. Misexpression in *Drosophila* of the mutant form of human torsinA, pan-neuronally or in monoaminergic cells results in a locomotor defect [6] and aberrant bouton morphologies at synapses [7], though the function of Dtorsin is unclear. Muraro and Moffat [8] have reported that targeted down-regulation of the *dtorsin* gene in the *Drosophila* eye caused progressive degeneration of the retina, but no loss-of-function mutants of *dtorsin* have previously been characterized.

To investigate the function of *dtorsin*, we generated loss-of-function mutant fly lines by the ‘ends-out’ gene targeting method in which homologous recombination at the ends of the gene results in the exchange of the entire targeted genomic sequence with a heterologous sequence [9], [10]. By replacing the *dtorsin* gene with unrelated sequence from the *white+* gene, we created a complete deletion of the *dtorsin* gene in the genome.

Mutant flies showed the following phenotypes: 1) the vast majority of mutant males died by the pre-pupal stage; 2) the few surviving adult males were sterile with reduced pigmentation and thin, short bristles; 3) the late third instar male larvae exhibited locomotor deficits. As some of these phenotypes are associated with mutations that disrupt dopamine metabolism [11], [12], we explored this pathway in the mutant flies using both biochemical and genetic approaches. Our findings indicate that disrupted dopamine metabolism is at least one consequence of this null mutation and that *dtorsin* is a novel positive-regulator of GTPCH protein in *Drosophila*.

## Results

### Generation of *dtorsin* loss-of-function mutants by homologous recombination

To investigate the function of *dtorsin*, we generated a loss-of-function mutation using ‘ends-out’ gene targeting designed to remove almost the entire coding sequence [9], [10] (Figure S1A and Materials and Methods). We screened approximately 40,000 flies and obtained fourteen lines that had a *white+* (*w^+^*) insertion on the X chromosome. Seven of the fourteen were semi-lethal lines, in which only a few adult males survived. PCR analysis with *dtorsin* gene-specific primers demonstrated that males from each of the seven semi-lethal lines lacked the *dtorsin* gene, while males of the seven viable lines retained the *dtorsin* gene (data not shown). The targeted homologous recombination event was expected to yield the same genomic structure in all correctly targeted lines. To confirm this, we performed Southern blot analysis on two semi-lethal lines, each derived from a different donor line (*y w dtorsin^KO13^*/Y males from B2-1 and *w dtorsin^KO78^*/Y males from B7-1; see Materials and Methods). The results were consistent with precise homologous recombination and replacement of the *dtorsin* gene with the targeting construct (Figure S1B). The integrity of the junctions was confirmed by sequencing of the PCR products. Both *dtorsin^KO13^* and *dtorsin^KO78^* lack all *dtorsin* sequence from the translation initiator ATG to 16 bases upstream of the termination codon (data not shown), leaving no potential to produce Dtorsin protein in these lines. Anti-Dtorsin antibody revealed a 38 kDa protein band, the predicted molecular weight of the Dtorsin protein, in wild type adult whole body extracts and head extracts (Figure S2 lanes 1 and 3), the corresponding *dtorsin^KO13^*/Y male mutant extracts showed no clear band of 38 kDa protein (Figure S2 lanes 2 and 4). The semi-lethality of all seven correctly targeted lines was rescued by introducing a 1.9 kb genomic fragment that contained the entire *dtorsin* gene (Table S1). These results indicate that all seven semi-lethal lines were *dtorsin* loss-of-function mutants.

### Adult phenotype of the *dtorsin* mutants

**Figure 1:**
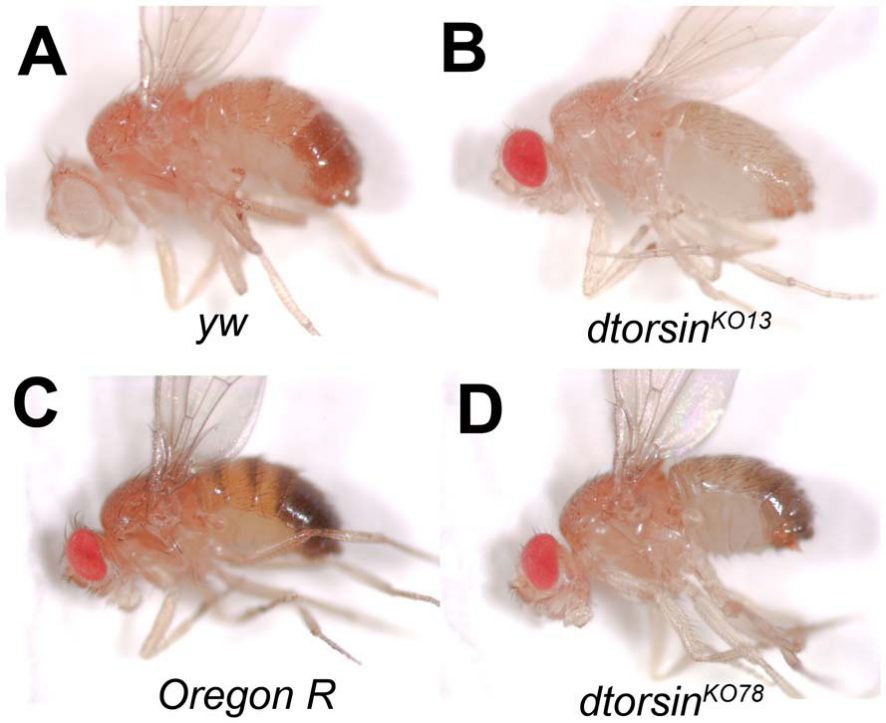
*dtorsin* loss-of-function mutant flies exhibit pigmentation phenotypes. **A–D,** Pigmentation phenotype of *dtorsin* mutants. Adult males of the genotype *y w*/Y (**A**), *y w dtorsin^KO13^*/Y (**B**), *Oregon R* (**C**), and *w dtorsin^KO78^*/Y (**D**) flies. The pictures for **A–D** were taken one day after eclosion to ensure pigmentation reached the maximum level. Since *dtorsin^KO78^* male has *y+* genotype, it was compared to the Oregon R, which is also *y+*.

All seven mutant lines had identical phenotypes. They were recessive semi-lethal lines with only a few males reaching adulthood. As these males were sterile, it was not possible to generate homozygous mutant females. To determine the stage of male lethality, we made a *dtorsin^KO13^* line carrying the *FM7i ActGFP* X-chromosome balancer. Hemizygous *dtorsin* mutant male first instar larvae were identified by their GFP-negative phenotype. Most of these animals developed to late third instar larvae, and died at the pre-pupal stage. Some mutants entered pupation but failed to eclose. In isolated cultures of hemizygous *dtorsin*-null larvae, about 10% of these developed to the adult stage, while 94% of wild type (*y w*) larvae developed to the adult stage when maintained under the same conditions. In mixed cultures where larvae of both genotypes were present, very few *dtorsin* null males developed to the adult stage (Table S1). The few surviving male adults showed the following characteristic phenotypes. The body color appeared paler than wild type, which suggested reduced pigmentation of the cuticle (Figure 1A, B, C, and D). The flies had thin, short bristles (Figure S3A and B), moved slowly, and, as noted above, were sterile. All of these phenotypes were rescued by introducing the 1.9 kb genomic fragment that contained the coding region of the *dtorsin* gene (Table S1 and Figure S3C). UAS-*dtorsin* cDNA driven by the Actin5c- or the Tubulin-GAL4 driver also rescued all phenotypes (Table S1).

### Mutant third instar larvae show a locomotion deficit

The surviving mutant adult flies moved slowly, compared to wild type. Since most of the mutants died at the pre-pupal stage, we studied the movements of the mutants as larvae prior to this stage. *Drosophila* larval crawling entails repeated, rhythmic, peristaltic contraction. During each peristaltic stride, muscle contractions are propagated from one end of the body to the other, passing through all eleven segments, one at a time [13]. Late third instar larval locomotion has been established in several recent studies as an excellent system to study the regulation of locomotion and has been used to evaluate various parameters of larval movement [13], [14], [15], [16], [17] and to examine the effects of various mutants on locomotion [13], [15], [16], [17], [18].

**Figure 2:**
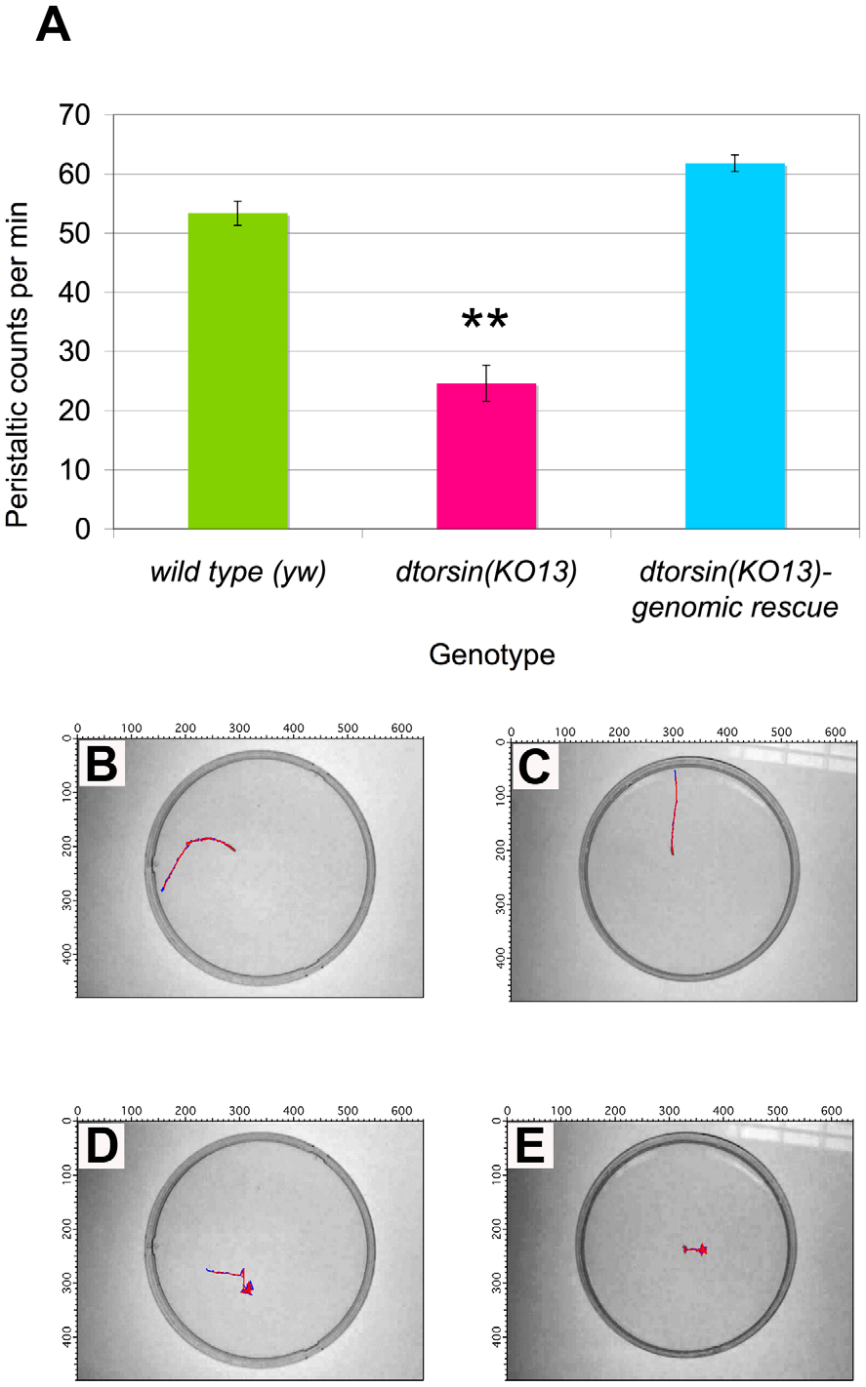
*dtorsin* mutant larvae have mobility defects. **A.** *dtorsin* mutants have reduced larval mobility. Peristaltic frequencies were counted manually during the wandering stage of third instar larvae of the genotype *y w* (n = 33), *y w dtorsin^KO13^*/Y (n = 29), and *y w dtorsin^KO13^*/Y; GDT101-2 (n = 21). Results are the mean ± S.E.M. ** p<0.0001, very significant difference between the wild type and the *dtorsin* mutant. **B–E.** Representative crawling patterns of the wild type (**B**, **C**) and the *dtorsin* mutant male (**D, E**) larvae. Locations of larval heads (blue line) and tails (red line) were captured by video recording for 60 seconds and visualized by the Prairie Dog software. Tracking of larval movement was stopped when the larva reached the edge of the plate. The genotypes of larvae were *y w* (**B, C**) and *y w dtorsin^KO13^/Y* (**D, E**). Most of the wild type larvae reached the edge of the dish within 30–40 seconds when they started from the center of the dish (**B, C**). Many of the *dtorsin* mutant male larvae circled around the same location without much net distance gain from the starting point (**D, E**). The numbers on x- and y- axes are arbitrary numbers indicating x- and y- coordinates to define locations of the larval head and tail.

To quantify the difference in locomotion between wild type and mutant larvae, we analyzed peristaltic frequency [13] allowing third instar male larvae to crawl freely on an agarose substrate in the absence of any obvious sensory cues for a period of 60 seconds. The entire period was recorded under high magnification with a camera video program. Whereas wild type (*y w*) male larvae moved at 53.3±2.1 strides per minute (n = 33), *dtorsin^KO13^* male larvae exhibited approximately a ∼50% decrease in stride frequency, 24.6±3.0 (n = 29, p<0.0001) (Figure 2A, and Video S1 and S2). The locomotion deficit in mutant male third instar larvae was fully rescued (61.8±1.4, n = 21) by introducing the genomic DNA fragment containing the entire *dtorsin* gene (Figure 2A).

Next we examined the crawling pattern of the larvae. To visualize the locomotion of larvae, the videos were analyzed with a newly developed program “Prairie Dog”. Wild type male larvae moved in a relatively straight line, with few changes in direction until they quickly reached the edge of the dish (Figure 2B, C). However, mutant male larvae frequently stopped and changed direction. As a result, mutant larvae often circled around the same location, gaining little distance from the starting point (Figure 2D, E).

### Neural expression of *dtorsin* can rescue the larval locomotion deficit

**Figure 3:**
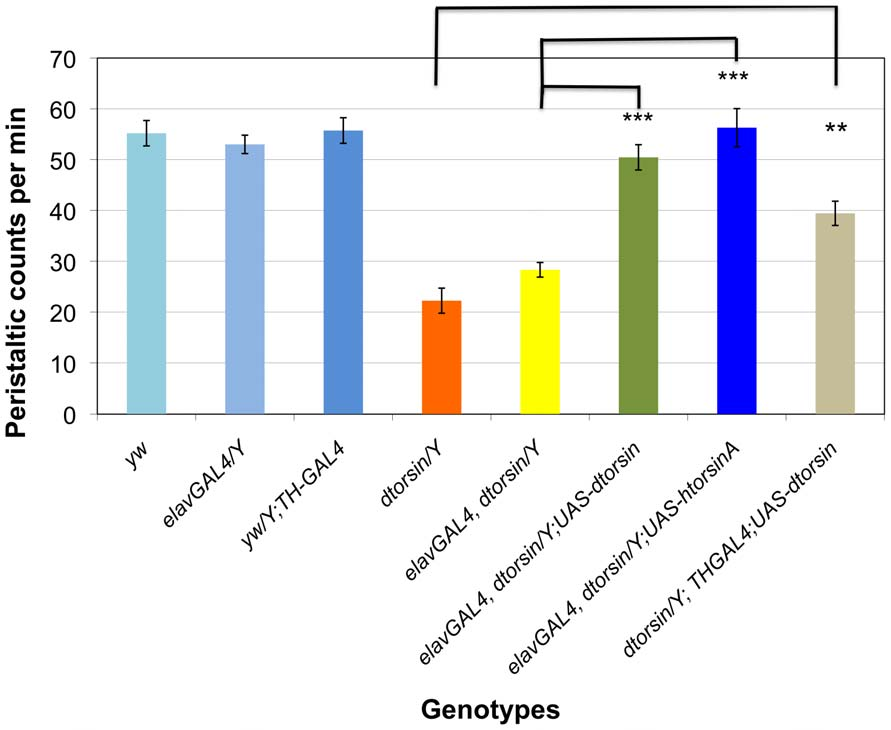
Rescue of *dtorsin* mutant male larvae mobility defects by cDNA expression. Peristaltic frequencies were counted for the wandering stage third instar larvae of the genotype wild type *y w*/Y male (n = 15), *w elavGAL4*/Y (n = 9), *y w*/Y; TH-GAL4(III) (n = 12), *w elavGAL4 dtorsin^KO13^*/Y (n = 32), *w elavGAL4 dtorsin^KO13^*/Y; UAS-*dtorsin*B5(II)/+ (n = 20), *w elavGAL4 dtorsin^KO13^*/Y; UAS-htorA#8(II)/+ (n = 20), *y w dtorsin^KO13^*/Y (n = 28), and *y w dtorsin^KO13^*/Y; TH-GAL4(III)/+; UAS-*dtorsin*B5(II)/+ (n = 12). Results are mean ± S.E.M. *** p<0.0001, very significant difference between the *dtorsin* mutant male (*w elavGAL4 dtorsin^KO13^*/Y) and the *dtorsin* mutant male with elavGAL4/UAS-*dtorsin*. *** p<0.0001, very significant difference between the *dtorsin* mutant male (*w elavGAL4 dtorsin^KO13^*/Y) and the *dtorsin* mutant male with elavGAL4/UAS-htorA. ** p<0.001, significant difference between the *dtorsin* mutant (*y w dtorsin^KO13^*/Y) and the *dtorsin* mutant male with TH-GAL4/UAS-*dtorsin*.

To determine whether the larval mobility deficit was specifically due to lack of Dtorsin in neurons, we used the GAL4/UAS system [19] to express a *dtorsin* cDNA using neuronal drivers (Figure 3). When the pan-neuronal driver elavGAL4 was combined with the UAS-*dtorsin* cDNA transgene, a strong rescue of the larval mobility was observed (50.5±2.5, n = 14, p<0.0001) compared to the equivalent *dtorsin*-null male larvae (elavGAL4, *dtorsin^KO13^*) lacking the UAS-*dtorsin* transgene (28.3±1.4 strides/min, n = 32). With the dopaminergic tissue-specific driver TH-GAL4 and the UAS-*dtorsin* transgene, we observed a significant increase of peristaltic frequency (39.4±2.4, n = 12, p = 0.0003) compared to the control (*y w dtorsin^KO13^*), although the rescue was weaker than that achieved with elavGAL4, suggesting that other neurons in addition to dopaminergic neurons may play important roles in regulating larval mobility in *dtorsin* null mutants. Transgenes of elavGAL4 or TH-GAL4 alone had no effect on the mobility of larvae with or without the *dtorsin^KO13^* null mutation (Figure 3). Expression of the *dtorsin* cDNA controlled by a muscle-specific driver, MHC-GAL4 [20] did not rescue the *dtorsin* null mutant larval mobility defect (data not shown). These results suggested that *dtorsin* expression in neurons plays an important role in regulating larval mobility.

### Rescue of larval locomotion deficit by neuronal expression of human torsinA

Next, we examined if the wild type human torsinA cDNA could rescue the *dtorsin* null mutant mobility defect. We found that human torsinA cDNA expressed with the Actin5c- or the Tubulin-GAL4 drivers rescued all phenotypes (pigmentation, bristle, and mobility) at very low frequency (Table S1). When this same human torsinA was expressed with the neuron-specific driver elavGAL4, a very significant rescue of the 3rd instar *dtorsin* null male larval mobility was observed (56.3±3.8 strides/min, n = 14, p<0.0001) compared to the control *dtorsin* null male larvae (28.3±1.4 strides/min, n = 32) (Figure 3). The peristaltic frequency of the *dtorsin* null male larvae that expressed the human torsinA cDNA in neurons was statistically similar to the wild type control (*y w*; 55.2±2.5 strides/min, n = 15). These data indicate that the neuronal functions of the human torsinA and *dtorsin* are very well conserved with respect to the regulation of the larval locomotion.

### Dopamine restored locomotor deficit phenotype in *dtorsin* mutant lines

In *Drosophila*, ingestion of dopamine increases dopamine pools in the fly head, though in mammals peripheral dopamine does not enter into the brain [21]. Since expression of *dtorsin* in dopaminergic cells produced detectable rescue of the locomotion phenotype, we tested whether addition of dopamine to fly food could also rescue this defect. Previous studies have shown that dopamine, serotonin, and octopamine can all affect larval locomotion as well as adult behaviors in *Drosophila* [16], [22], [23]. Consequently, we also tested whether serotonin or octopamine could rescue the locomotion deficit of male *dtorsin* null larvae.

**Figure 4:**
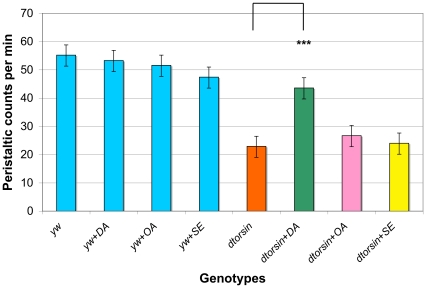
Rescue of *dtorsin* mutant larvae mobility defects by dopamine feeding. Peristaltic frequencies were counted for the wandering stage third instar larvae of the genotype wild type (*y w*) (n = 15), wild type (*y w*) (+20 mM dopamine) (n = 10), wild type (*y w*) (+20 mM octopamine) (n = 8), wild type (*y w*) (+10 mM serotonin) (n = 9), *y w dtorsin^KO13^*/Y (n = 28), *y w dtorsin^KO13^*/Y (+20 mM dopamine) (n = 15), *y w dtorsin^KO13^*/Y (+20 mM octopamine) (n = 10), and *y w dtorsin^KO13^*/Y (+10 mM serotonin) (n = 13). Results are mean ± S.E.M. *** p<0.0001, very significant difference between the *dtorsin* mutant with no drug supplementation and the *dtorsin* mutant with 20 mM dopamine supplementation. There was no significant difference between the *dtorsin* mutant males with no drug supplementation and the *dtorsin* mutant males with 20 mM octopamine (p = 0.4240) or the *dtorsin* mutant male with 10 mM serotonin (p = 0.8148). There was no significant difference between the wild type with no drug supplementation and the wild type with drug (+20 mM dopamine, +20 mM octopamine, or +10 mM serotonin).

Adding 20 mM dopamine in the food during the larval stage significantly increased the stride frequency of male *dtorsin^KO13^* mutant larvae (43.6±3.2, n = 15, p<0.0001) compared to that of the *dtorsin^KO13^* larvae grown without drug supplementation (22.9±2.5, n = 28). However, no significant changes were detected in the mutant larvae grown with 20 mM octopamine (26.7±3.6, n = 10, p = 0.4240) or 10 mM serotonin (24.0±3.9, n = 13, p = 0.8148) supplementation. No significant differences were observed between wild type larvae after supplementation with dopamine (53.3±2.4, n = 10, p = 0.6135), octopamine (51.6±3.6, n = 8, p = 0.4028), or serotonin (47.4±2.8, n = 9, p = 0.0562) and those grown without monoamine supplementation (55.2±2.5, n = 15) (Figure 4).

**Figure 5:**
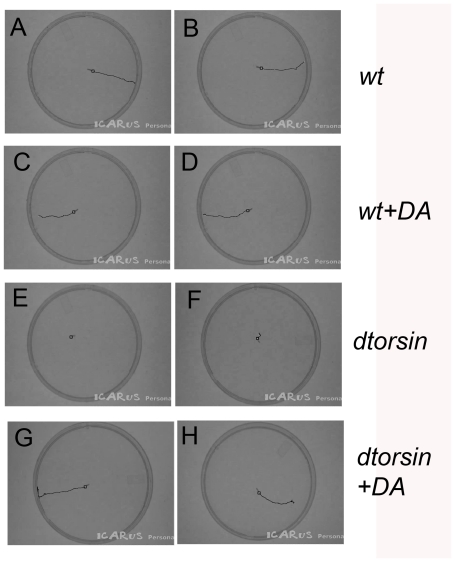
Effect of dopamine on the larval crawling patterns. Video tracking of the representative crawling patterns of the wild type (**A**, **B**), the wild type with 50 mM dopamine supplementation (**C**, **D**), the *y w dtorsin^KO13^* mutant male (**E**, **F**), and the *y w dtorsin^KO13^* mutant male with 50 mM dopamine supplementation (**G**, **H**). Locations of larval heads (black lines) were tracked by the ICARUS software. Tracking was stopped when the larva reached the edge.

After dopamine ingestion, the *dtorsin^KO13^* male mutant larvae also exhibited relatively straight crawling patterns, similar to those of the wild type larvae with or without dopamine (Figure 5). The reduced cuticular pigmentation phenotype of surviving male adults was also partially rescued by dopamine supplementation (Figure S4).

### Dopamine level is significantly reduced in *dtorsin* mutant heterozygotes

**Figure 6:**
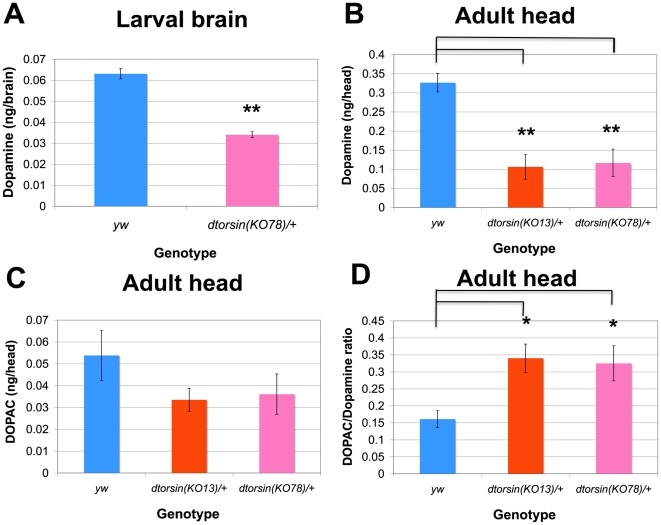
Effect of *dtorsin* mutation on dopamine in larval brains and adult heads. **A**. Effect of *dtorsin* heterozygous mutation on dopamine pools in larval brains (nanograms per brain). Monoamines were extracted from third instar larval brains and dopamine was separated and quantified by HPLC. *dtorsin* mutation significantly reduced dopamine levels. The genotypes of larvae were *y w* females (n = 3 independent replications) and *w dtorsin^KO78^*/+ females (n = 3 independent replications). Error bars indicate S.E.M. ** p<0.001, significant difference between the wild type and the *dtorsin* heterozygous mutant. **B**.-**D**. Effect of *dtorsin* heterozygous mutation on dopamine levels (nanograms per head). (**B**), DOPAC levels (nanograms per head) (**C**), and DOPAC/dopamine ratio (**D**) in adult heads. The genotypes of adults were *y w* females (n = 3 independent replications), *y w dtorsin^KO13^*/+ females (n = 3 independent replications), and *w dtorsin^KO78^*/+ females (n = 3 independent replications). Error bars indicate S.E.M. ** p<0.001, significant difference in dopamine levels between the wild type and the *dtorsin* heterozygous mutant females (**B**). No statistically significant difference was observed in DOPAC levels between the wild type and the *dtorsin* heterozygous mutant females, although the mutant levels were slightly lower (**C**). * p<0.01, significant difference in DOPAC/dopamine ratio between the wild type females and the *dtorsin* heterozygous mutant females (**D**).

In *Drosophila*, dopamine metabolism has a fundamental role in pigmentation of the cuticle [11], [12], and in many neurologically-mediated processes, including locomotion [24]. Consequently, as the reduced pigmentation and the locomotion deficits in the *dtorsin* mutants and their rescue by ingestion of dopamine suggested that dopamine metabolism must be disturbed by the *dtorsin* mutation, we measured dopamine levels in the third instar larval brain and adult heads. Since large numbers of adults or larvae were required for the analyses of dopamine by HPLC (see Materials and Methods), we used heterozygous mutants for these experiments. Wild type (*y w*) female larvae contained 0.0631±0.0025 ng dopamine/brain (n = 3, 75 brains per assay), whereas *dtorsin* heterozygous female larvae (*dtorsin^KO78^/+*) had 0.0340±0.0014 ng dopamine/brain (n = 3) corresponding to a 46% reduction (p<0.001) (Figure 6A). Dopamine pools in the heads of adult female heterozygotes were even more dramatically altered, with dopamine levels reduced from 0.33 ng/head in the *y w* control flies to approximately 0.10 ng/head in two different *dtorsin* mutants (*dtorsin^KO13^*/+ : 0.11±0.02 ng/head, n = 3, p<0.001; *dtorsin^KO78^*/+ : 0.12±0.04 ng/head, n = 3, p<0.001) (Figure 6B).

We next sought to determine whether trafficking of dopamine was affected by the *dtorsin* mutation. Turnover of dopamine during synaptic release can be inferred by the levels of the dopamine metabolite 3,4-dihydroxyphenylacetic acid (DOPAC) and by the ratio of DOPAC to dopamine in flies, as in mammals [21], [25]. Although the levels of DOPAC in the *dtorsin* heterozygous mutant were decreased, the reduction was clearly not proportional to the reduction in dopamine levels. In consequence, the resulting DOPAC:dopamine ratios were elevated over two-fold (Figure 6C, D). These neurochemical data suggested that the reduction of *dtorsin* in dopaminergic cells may lead to depressed synthesis of dopamine as well as trafficking abnormalities. Alternatively, the loss of *dtorsin* might lead to loss of dopaminergic cells.

**Figure 7:**
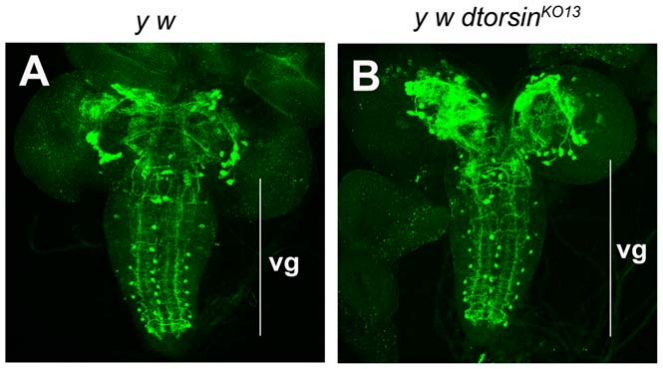
The third instar larval brains of *dtorsin* mutants have TH-positive cells. **A** and **B.** Comparison of patterns of TH immunostaining (in green) in the third instar larval central nervous system of the wild type male (**A**) and the *dtorsin* mutant male (**B**). **A**: A projection of twenty-seven confocal z-sections of wild type (*y w*) third instar male brain is shown. **B**: A projection of thirty confocal z-sections of *y w dtorsin^KO13^*/Y third instar male larval brain is shown. The projection was made from a series of confocal Z-sections (optical thickness 2 µm) that covered the whole brain regions using the NIH Image-J program with the Max intensity method. vg: ventral ganglion.

Tyrosine hydroxylase (TH) catalyses the first and rate-limiting step in the dopamine biosynthesis pathway and is expressed in all dopaminergic neurons. Therefore, we next examined third instar larval brain using anti-dTH antibody to determine whether *Drosophila* TH (dTH) is expressed in dopaminergic neurons in the brains of *dtorsin* null mutants [26]. The dopaminergic cell pattern has been well characterized in the central nervous system of *Drosophila* larvae and consists of four clusters of 4–10 neurons in each brain hemisphere and a stereotyped pattern of three to five paired or unpaired neurons per segment in the ventral ganglion [27] (Figure 7A). We found that dTH was expressed in the brains of hemizygous *dtorsin^KO13^* third instar larvae and that the number of dTH-positive cell bodies was approximately normal and clearly not sufficiently reduced to account for the dramatic loss of dopamine observed. However, we did observe some apparent morphological changes in the positioning of cell bodies of dTH-positive neurons and in the patterning of dTH-positive axons in the central brain region of the hemizygous *dtorsin* null male larvae (Figure 7B). Further studies are underway to understand the exact nature of these structural differences between the wild type and the *dtorsin* null mutant brains, but we are able to conclude that loss of Dtorsin expression and the consequent reduction in dopamine levels are not associated with failure of dopaminergic neurons to differentiate or their subsequent degeneration.

### Genetic interactions of *dtorsin* with *Pu* (*Punch*) or *ple* (*pale*)

Dopamine synthesis is directly regulated by the specific activity of TH, which, in turn, is modulated by the amount of its cofactor, tetrahydropterin (BH4). In consequence, TH activity is regulated indirectly by the activity of the rate-limiting enzyme in BH4 synthesis, GTP cyclohydrolase (GTPCH) in flies, as in mammals [28], [29]. We addressed the hypothesis that dopamine synthesis is affected by mutations in *dtorsin* and further pursued a putative role in dopamine trafficking by first conducting gene interaction analysies. We tested for genetic interaction of the null *dtorsin^KO13^* allele with mutant alleles of *ple* (*pale*), which encodes TH [30], [31], *Pu* (*Punch*), the *Drosophila* ortholog of GTP cyclohydrolase (GTPCH) [32], and *DAT*, encoding the dopamine transporter [33]. *Pu* and *ple* loss-of-function mutants show similar phenotypes to each other [34], as *Pu* mutants exhibit reductions in dopamine pools due to limiting BH4 levels [35]. Homozygous *Pu* loss-of-function mutants die during late embryogenesis with completely unpigmented and weak cuticles [32]. The human orthologs of these two genes underlie DYT5 and DYT14 (Dopa-responsive) dystonia [1] which is phenotypically similar to DYT1 dystonia, except that DYT5 and DYT14 patients respond very well to the administration of L-dopa [36], [37] whereas DYT1 patients do not. Most DYT14 patients have dominantly inherited loss of function mutations in the *GCH1* gene, while mutations in the *TH* gene are responsible for a rare recessive form [36]. The third gene involved in dopamine metabolism, *DAT*, was found to be mutated in a hyperactive fly line, *fumin*. Elimination of DAT activity in the *DAT^fumin^* mutant caused a dramatic increase in the length of the waking phase [33].

**Figure 8:**
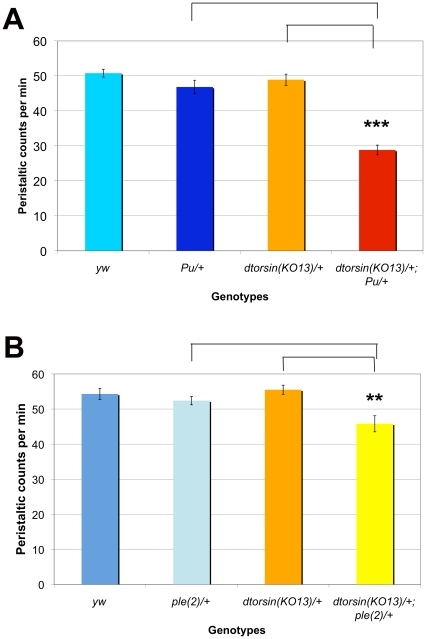
The double heterozygous combinations for *dtorsin* and *Punch* or *pale* mutants show reduced mobility in third instar larvae. **A.** Peristaltic frequencies were counted for the wandering stage third instar larvae of the genotype *y w* female (n = 31), *Pu^Z22^*/+ female (n = 23), *dtorsin^KO13^*/+ female (n = 22), and *dtorsin^KO13^*/+; *Pu^Z22^*/+ female (n = 30). Results are the mean ± S.E.M. **p<0.0001, significant difference between the wild type females (*yw*, *Pu^Z22^*/+, or *dtorsin^KO13^*/+) and the double heterozygous females (*dtorsin^KO13^*/+; *Pu^Z22^*/+). No significant difference was observed between the wild type females (*y w*) and the single heterozygous females (*Pu^Z22^*/+ or *dtorsin^KO13^*/+). **B.** Peristaltic frequencies for the wandering stage third instar larvae of the genotype *y w* female (n = 20), *ple^2^*/+ female (n = 20), *dtorsin^KO13^*/+; female (n = 31), and *dtorsin^KO13^*/+; *ple^2^*/+ female (n = 30). Results are the mean ± S.E.M. *p<0.05, significant difference between the wild type females (*y w*, *ple^2^*/+, or *dtorsin^KO13^*/+) and the double heterozygous females (*dtorsin^KO13^*/+; *ple^2^*/+). No significant difference between the wild type females (*y w*) and the single heterozygous females (*ple^2^*/+ or *dtorsin^KO13^*/+).

In order to test the possibility of a genetic interaction between the *dtorsin* null mutation and loss-of-function mutations of *Pu*, *ple*, or *DAT^fumin^*, we analyzed the peristaltic stride frequency of the double-heterozygous female mutant larvae. No phenotype was seen in any single heterozygous mutant line. The stride frequencies of *Pu^Z22^*/+ males (48.7±2.0, n = 20) and females (46.8±1.9, n = 23) were statistically similar to the stride frequency of the *dtorsin^KO13^*/+ females (48.9±1.6, n = 22). However, the double heterozygous *dtorsin^KO13^*/+; *Pu^Z22^*/+ females showed very significantly reduced peristaltic frequency (28.8±1.4, n = 30, p<0.0001), relative to either single heterozygote (Figure 8A). While *dtorsin^KO13^*/*Y* male third instar larvae were mostly viable, very few *dtorsin^KO13^*/*Y*; *Pu^Z22^*/+ male of the third instar larvae were observed.

There was a much weaker interaction between *dtorsin* and *ple*. While no decreased locomotion phenotype was seen in *ple^2^*/+ females (52.4±1.2, n = 20), *dtorsin^KO13^*/+; *ple^2^*/+ female larvae showed a slight but significant reduction of peristalsis frequency (45.8±2.3, n = 31, p = 0.0007), compared to *dtorsin^KO13^*/+; *+*/+ females (55.5±1.3, n = 20) (Figure 8B).

No statistically significant interaction was detected between *dtorsin* and *DAT^fumin^*. The double heterozygous mutant *dtorsin^KO13^*/+;*DAT^fumin^*/+ females showed similar peristaltic frequency (46.1±1.4, n = 14, p = 0.5402) compared to single heterozygous mutant *dtorsin^KO13^*/+ females (47.6±2.9, n = 7) (Figure S5). The *DAT^fumin^* mutation also had no effect on the mobility of *dtorsin* hemizygous mutant males. The *DAT^fumin^* heterozygous mutant *dtorsin^KO13^*/Y; *DAT^fumin^*/+ males had a peristaltic frequency (21.6±3.6, n = 12, p = 0.7613) that was statistically identical to *dtorsin* mutant *dtorsin^KO13^*/Y males (22.9±2.5, n = 28).

Thus the *dtorsin* null mutation showed a very strong genetic interaction with the *Punch* (GTPCH) mutation, a weaker interaction with the *ple* (TH) mutation, and no detectable interaction with the *fumin* (DAT) mutation.

### GTPCH, but not TH activity, is severely reduced in *dtorsin* heterozygous mutant lines

**Figure 9:**
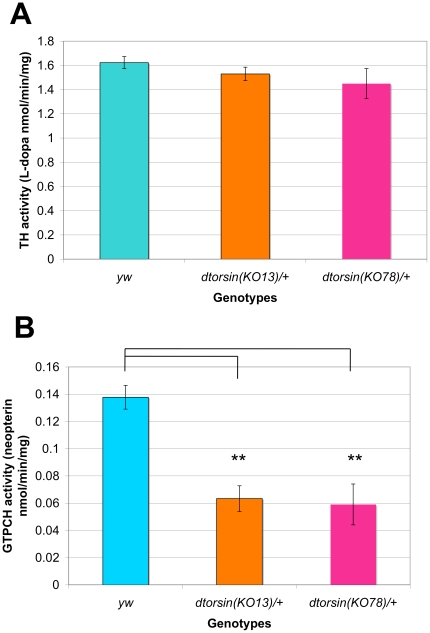
Effect of *dtorsin* mutation on TH activity and GTPCH activity in adult heads. **A**. Effect of *dtorsin* heterozygous mutation on TH activity in adult heads (L-dopa nmoles/min/mg protein). The genotypes of adults were *y w* (n = 3 independent replications), *y w dtorsin^KO13^*/+ (n = 3 independent replications), and *w dtorsin^KO78^*/+ (n = 3 independent replications). Error bars indicate S.E.M. No significant difference between the wild type and the *dtorsin* heterozygous mutant females. **B**. Effect of *dtorsin* heterozygous mutation on GTPCH activity (neopterin nmoles/min/mg protein). The genotypes of adults were *y w* (n = 3 independent replications), *y w dtorsin^KO13^*/+ (n = 3 independent replications), and *w dtorsin^KO78^*/+ (n = 3 independent replications). Error bars indicate S.E.M. ** p<0.01, significant difference in dopamine levels between the wild type females and the *dtorsin* heterozygous mutant females.

The above findings suggested that the Dtorsin function may regulate the *Pu* gene/GTPCH protein activity. To test this hypothesis, extracts were prepared from wild type (*y w*) and *dtorsin* heterozygous mutant (*y w dtorsin^KO13^*/+ and *w dtorsin^KO78^*/+) adult heads and TH and GTPCH activities were compared (Figure 9). No significant difference was detected between the TH activities of the *dtorsin* null heterozygous flies (*y w dtorsin^KO13^*/+ : 1.53±0.06 L-dopa nmoles/min/mg protein, n = 3 independent assays, p = 0.2962; *w dtorsin^KO78^*/+ : 1.45±0.12 L-dopa nmoles/min/mg protein, n = 3 independent assays, p = 0.2657) and the wild type flies (*y w*) (1.62±0.05 L-dopa nmoles/min/mg protein, n = 3 independent assays). However, a significant reduction in GTPCH activity was observed in the *dtorsin* null mutant heterozygous flies (*y w dtorsin^KO13^*/+ : 0.0633±0.009 neopterin nmoles/min/mg protein, n = 3 independent assays, p<0.01; *w dtorsin^KO78^*/+ : 0.0590±0.015 neopterin nmoles/min/mg protein, n = 3, p<0.01) compared to wild type flies (*y w*) (0.138±0.087 neopterin nmoles/min/mg protein, n = 3 independent assays). These results suggest that the *dtorsin* is a novel positive-regulator of GTPCH activity in *Drosophila*.

### GTPCH protein levels are severely reduced in *dtorsin* heterozygous and hemizygous mutant lines

**Figure 10:**
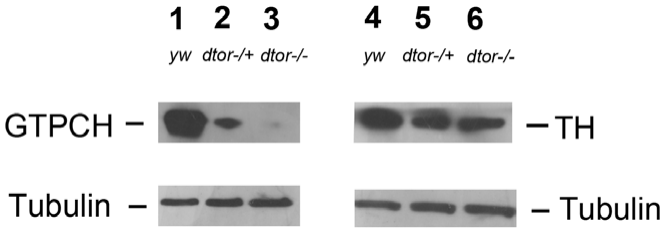
Effect of *dtorsin* mutation on TH and GTPCH protein levels. Total adult head extracts from the wild type males (*y w*) (lanes 1 and 4), *y w dtorsin^KO13^*/+ females (lanes 2 and 5), or *y w dtorsin^KO13^*/Y males (lanes 3 and 6) were analyzed by Western blots. The membrane was probed with anti-GTPCH B/C (lanes 1–3, upper panel) and reprobed with anti-Tubulin (lanes 1–3, lower panel). The second membrane was probed with anti-TH (lanes 4–6, upper panel) and reprobed with anti-Tubulin (lanes 4–6, lower panel). The location of GTPCH (43–45 kDa), TH (58 kDa), and alpha-Tubulin (50 kDa) are indicated. Twenty µg of proteins were loaded in each lane.

The above findings suggested that *dtorsin* might be required for maintaining GTPCH protein levels. To test this hypothesis, we prepared extracts from heads of wild type adult males (*y w*), *dtorsin* heterozygous adult females (*y w dtorsin^KO13^*/+), and *dtorsin* hemizygous adult males (*y w dtorsin^KO13^*/Y) and compared TH and GTPCH protein levels by Western blot analysis (Figure 10, lanes 1–3). No significant difference was detected between the TH protein levels of *dtorsin* null hemizygous flies (*y w dtorsin^KO13^*), heterozygous flies (*y w dtorsin^KO13^*/+ females), and of wild type flies (*y w*) (Figure 10, lanes 4–6). However, a very severe reduction in the level of GTPCH protein was observed in the *dtorsin* null mutant heterozygous females (*y w dtorsin^KO13^*/+) compared to wild type (*y w*). Moreover, only very low levels of GTPCH protein level were detected in *dtorsin* null mutant hemizygous males, suggesting only a very small amount of GTPCH proteins are present in the hemizygotes (Figure 10, lanes 1–3). These results support the conclusion that *dtorsin* is required for maintaining the level of GTPCH protein in *Drosophila* though the molecular mechanism by which it acts remains to be elucidated.

## Discussion

The human genome contains four paralogous genes of the torsin family, while *Drosophila* has a single torsin gene, *dtorsin*, encoding a protein of the AAA family of adenosine triphosphatases (ATPases), related to the Clp protease/heat shock family [4]. The precise functions of these genes and the degree to which their activities may overlap are not known, though *TOR1A* is notable for causing early-onset dystonia due to a codon deletion that removes a particular glutamic acid residue from the torsinA protein. TorsinA-null knock-out mice (*Tor1a* ^−*/*−^) have been created and die at birth with abnormal nuclear membrane morphology in neural cells [38]. In comparison to the human genes, *dtorsin* is most closely related to *TOR1A* [4], [39], suggesting that investigation of the fly protein's function may be informative concerning the pathway(s) disrupted in human early onset dystonia. Our generation and characterization of a null mutant for *dtorsin* revealed that it is an essential gene in flies, as mutants typically fail to reach adulthood and those that do survive fail to reproduce. Null *dtorsin* mutant flies display both morphological and behavioral phenotypes that may provide starting points for elucidating activities of the Dtorsin protein, using both biochemical and genetic strategies. Our initial examination of these phenotypes indicates that at least one major disruption in *dtorsin* mutant flies involves regulation of dopamine, a function also implicated in human dystonia [40]. We expect that the availability of the *dtorsin* null mutant will facilitate investigation of this activity and other effects of torsin deficiency that are likely to emerge with more detailed study.

We were led to examine dopamine metabolism in the *dtorsin* mutants because of the phenotypes exhibited by rare adult survivors, which had a pale body color and thin bristles (Figure 1 and S3), moved slowly and were sterile. A series of reports have shown that dopamine not only functions as a neurotransmitter but also plays a role in cuticle pigmentation, fertility, and other vital functions in *Drosophila* [11], [12], [41]. Thus the most evident phenotypes exhibited by the *dtorsin* null mutants are consistent with previously reported phenotypes caused by dopamine deficiency.

Disruption of dopamine metabolism in the *dtorsin* mutants was supported by HPLC analysis of heterozygous *dtorsin* mutant larval and adult brains/heads which demonstrated a significantly reduced dopamine level (Figure 6) and by rescue of the larval locomotion defect and cuticle pigmentation by feeding dopamine (Figure 4, 5, S4). Thus, dopamine synthesis appears to be disrupted in *dtorsin* null flies and is likely to be largely responsible for both the deficit in larval locomotion and in cuticle pigmentation. While ingestion of serotonin and octopamine had no significant effect on the *dtorsin* larval locomotion deficit, the results of this particular assay do not exclude the possibility that other monoamine pathways may also be altered in these flies. Indeed the weaker rescue of the locomotor phenotype by expression in dopaminergic neurons than by more widespread neuronal expression (Figure 3) suggests a role for other neuronal elements/pathways. Further studies, including the measurement of serotonin and octopamine pools, will be required to examine the effect of *dtorsin* null mutation on these particular pathways.

Our visualization of dopaminergic neurons in mutant flies indicated that the reduced dopamine levels were not due to reduced numbers of dopaminergic neurons (Figure 7), suggesting a relative decrease in dopamine production in the absence of Dtorsin. Dopamine synthesis involves hydroxylation of the amino acid L-tyrosine by TH to produce 3,4-dihydroxy-1-phenylalanine (L-dopa), which is subsequently decarboxylated by aromatic-L-amino acid decarboxylase to form dopamine. TH activity depends on the availability of its cofactor, BH4 [32], which is produced by conversion of GTP to 2,4-dihydroneopterin triphosphate by GTPCH, followed by reduction and dephosphorylation to the final product, BH4, by 6-pyruvoyltetrahydropterin synthase and sepiapterin reductase, respectively [29].

In flies, TH is encoded by the *ple (pale)* gene and GTPCH is produced from the *Pu (Punch)* gene. Our finding that genetic interaction of mutations in *dtorsin* and *Pu* produces a strong defect in the larval locomotor assay whereas genetic interaction of *dtorsin* and *ple* produces a weaker but notable effect (Figure 8), is consistent with the view that dopamine metabolism is further compromised in these double mutants. This genetic effect parallels our biochemical measurement of a severe reduction of GTPCH activity but not TH activity, in *dtorsin* heterozygous adult flies (Figure 9). Furthermore, western blot analyses demonstrated that GTPCH protein levels are severely reduced in *dtorsin*-null heterozygotes and hemizygotes (Figure 10). Taken together, our experiments suggest that presence of functional Dtorsin is required to maintain GTPCH protein levels, indicating that, through an as yet undetermined mechanism, *dtorsin* is a positive-regulator of GTPCH protein and dopamine synthesis in *Drosophila*.

Interestingly, GTPCH is also critical for the activity of other aromatic acid hydroxylases, nitric oxide synthatase [29], and alkylglycerol monooxygenase [42]. Thus GTPCH activity is also essential to the serotonin synthesis, suggesting that it too may be disrupted in the *dtorsin* mutant. Why then did addition of serotonin have no effect on the larval mobility in our experiments? There are two possible explanations. The first is that the larval mobility assay that we used may be sensitive only to a defect in the dopamine pathway. The second is that the TH protein has a much lower affinity for BH_4_ compared to tryptophan hydroxylase, the rate-limiting enzyme in the serotonin synthesis [43], so that TH activity is preferentially affected when the concentration of BH4 is decreased to submillimolar levels. Further studies will be needed to distinguish these alternative possibilities.

Notably, human GTPCH and TH are defective in two forms of dopa-responsive dystonia (DYT14 and DYT5, respectively), while torsinA is altered in DYT1 dystonia. Deficits in dopamine neurotransmission have been implicated in all these forms of dystonia that have a similar clinical appearance, manifesting as early onset generalized dystonia [37], [40]. DYT5 and DYT14 dystonia results from mutations in genes needed for biosynthesis of dopamine, and symptoms can be alleviated with L-DOPA [36]. In the case of DYT1 dystonia, the abnormality seems to involve insufficient neurotransmission by dopamine, although levels of dopamine are normal and, in this case, L-DOPA is not therapeutic. In DYT1 patients, the number of dopamine receptor binding sites in the striatum are reduced [44], [45], and, in addition, neuroleptic medications which block D2 receptors can lead to dystonia reactions and tardive dystonia [46]. These studies support a link between *TOR1A* mutation, altered dopaminergic neurotransmission and dystonic symptoms. This concept has been supported in a variety of DYT1 mouse models showing that expression of mutant torsinA causes an increase in dopamine turnover [47], a decrease of release and uptake of dopamine in response to amphetamine [48], [49] or electrical stimulation [50], and impairment of dopamine D2 receptor function [51]. So, although the mechanism through which dysfunctional torsinA leads to compromise of dopaminergic neurotransmission is not clear, many lines of evidence supports this connection [2], [40].

It is important to stress that the mutated abnormal torsinA protein is expressed together with normal torsinA in the DYT1 animal models and DYT1 patients, while the null *dtorsin* fly lines do not express any functional Dtorsin protein. Thus, the fly mutant is fundamentally different from other DYT1 disease model systems. The interaction between the DYT1-mutant form of the torsinA protein (for example ΔGAG torsinA) and the wild type torsinA protein could be very important for the DYT1 disease manifestations, presumably leading to a decrease in wild type activity. Consequently, it is essential to understand the normal function of the torsinA protein in order to understand how compromise of its function leads to DYT1 dystonia. The availability of fly lines mutant for *dtorsin* provides the basis for applying the powerful tools of *Drosophila melanogaster* genetics to define the function(s) of the sole torsin family member in this species, with the expectation that these activities may provide insight into torsinA function in humans. In this regard, the ability of the human torsinA cDNA to rescue the mobility defect of the *dtorsin* null mutant demonstrates that at least the torsin function involved in regulation of larval locomotion, which requires expression in neurons, is well conserved between the fly and the human. The *dtorsin* null fly system will therefore permit analysis of the effects of various DYT1-causing mutations on this function, using the biochemical, pharmacological, and genetic approaches.

## Materials and Methods

### Fly stocks

Flies were kept on standard medium, except for larval locomotion analysis (see below). The *y w; P{70FLP}11 P{70I-SceI}2B Sco/CyO*, *S^2^* line for the gene targeting, GAL4 driver lines, and the *FM7i, P{w[+mC] = ActGFP}JMR3/C(1)DX, y f* were obtained from the Bloomington Stock Center. The MHC-GAL4 line was obtained from Dr. Aaron DiAntonio (Washington University School of Medicine, MO). The *DAT^fumin^* line was a kind gift from Dr. F. Rob Jackson (Tufts University School of Medicine, MA).

### Generation of the *dtorsin* loss-of-function mutant lines

The “ends-out” targeting scheme was a modified version of a method described previously [9], [10]. The donor vector was constructed using pw25 [10] (a gift from Dr. Kent Golic, University of Utah, UT) in two steps. In step 1, a 4 kb genomic fragment including the 5′ portion of *dtorsin* was amplified from genomic DNA (*y w* male) by PCR using the following primers: 5′- AGCGGCCGCGATATTATGACCCTGGCCAAGGGCTACTTGGAGCAGCTACCAGGACCGGGCAAGGCATCCTCCTATCGCT -3′ (TK4A) and 5′-CGGTACCGCAAGGAAAATTCCGCGGTGGAATGTATAGATGGGCAAAAGCGATAGGCAGCATAAGACTAGA-3′ (TK3A). The product was digested with *NotI* and *Acc65I* and cloned into the *NotI* and *Acc65I* sites of pw25.

In step 2, a 3.5 kb genomic fragment of the 3′ portion of *dtorsin* was amplified from genomic DNA by PCR using the following primers: 5′-AGGCGCGCCGGCCATGGCCATTTACTGAGAAAGGACCGACCGACTCCACATTTTAATGC -3′ (TK2) and 5′-CCGTACGCAATGCTGTATATATTAATCGTTTGAGTATAAACAAATATCACGACTTA-3′ (TK1). The product was digested with *AscI* and *BsiWI* and cloned into the *AscI* and *BsiWI* sites of the plasmid made in step 1. Nine fly lines carrying independent insertions of the donor construct were generated using standard procedures. Two independent lines, B2-1 (2nd chromosome insertion) and B7-1 (3rd chromosome insertion) were used for further experiments. Males heterozygous for each donor insertion were crossed to *y w; P[70FLP]11 P[70I-SceI]2B Sco/S^2^ CyO* females, and resulting larvae were heat-shocked at 38°C for 1 hour at 3 days after egg laying (AEL). The resulting unbalanced females were mated in groups of 10 to *y w; P[70FLP]11 P[70I-SceI]2B Sco/S^2^ CyO* males. The progeny from this cross were heat-shocked at 3 days AEL, and 405 vials were screened for red eye female upon eclosion. Non-mosaic, unbalanced flies were saved as FLP-insensitive integrations. In total 14 FLP-insensitive integrations were found that mapped to the X chromosome. Of those, seven lines were hemizygous viable and seven (*y w dtorsin^KO13^*, *y w dtorsin^KO14^*, *y w dtorsin^KO18^*, *y w dtorsin^KO22^*, *y w dtorsin^KO26^*, *y w dtorsin^KO38^*, and *w dtorsin^KO78^*) were semi-lethal. Few male adults survived from the seven semi-lethal lines, and no homozygous females survived.

### Southern blot analysis

Southern blots were performed using standard techniques. The DNA was prepared from adult male survivors of *y w dtorsin^KO13^*/Y and *w dtorsin^KO78^*/Y.

Two DNA fragments were generated to the 5′ and 3′ adjacent region of *dtorsin* coding sequence by PCR for radio labeled probes (Probe 1 and Probe 2) (Figure S1A) and gel purified. A 514 bp DNA fragment (Probe 1) was amplified by using the following primers: 5′- GGATCCACCCGTGGAGCCGGCGAAACTTTTCCGGGTGCAG -3′ and 5′- GGCCATGGCCATTTACTGAGAAAGGACCGACCGACTCCAC -3′ and detected a 1.5 kb *BamHI* fragment in wild type (*y w* and *w*) in genomic Southern blots (Figure S1B). A 381 bp DNA fragment (Probe 2) was amplified by using the following primers: 5′- GATTGCAGCACTTGCCGGCAACCGTTTCTATCGATAGTTG -3′ and 5′- GGATCCTGGACAGGATACGCTTGACGGATGGGCCTTGTTG -3′ and detected a 0.5 kb *BamHI* fragment in wild type (*y w* and *w*) (Figure S1B).

### Genomic rescue line

A 1.9 kb genomic fragment containing *dtorsin* was amplified from genomic DNA by PCR using the following primers: 5′- GCGGCCGCTTGGACCTGGACTTTTCGGGATCCTGGACAGGATACG -3′ and 5′-GAATTCCCTCAAAGATCTGGGACTAGACGGAAAGCAGAGCGATTT-3′. The fragment contains 0.4 kb 5′ non-coding region, the whole coding region of the *dtorsin* gene, and 0.37 kb 3′ non-coding region. The product was digested with *EcoRI* and *NotI* and cloned into the *EcoRI* and *NotI* sites of pCaSpeR3. Four fly lines (GDT101-1∼4) carrying independent insertions of the donor construct were generated using standard techniques. All four lines were crossed to individual *dtorsin* mutant lines.

### UAS lines

A 1.2 kb *dtorsin* cDNA was amplified from LD20854 (LD library, BDGP) by PCR using the following primers: 5′-CCGGAATTCCCTTGCATTTTATGATGAGCTTTCCACGCATGTTA-3′ and 5′-GTTGCGGCCGCCATTAAAATGTGGAGTCGGTCGGTCCTTTCTCA-3′. The product was digested with *EcoRI* and *NotI* and cloned into the *EcoRI* and *NotI* sites of pUAST [19].

The transgenic line B5 with UAS*-dtorsin* transgene on the second chromosome was used for the rescue experiments.

A 1.0 kb human torsinA cDNA was amplified from pcDNA3-htorA [52] by PCR using the following primers: 5′-GCGGGATCCATTCATGAAGCTGGGCCGGGCCGTGCTGGGCCTGC-3′ and 5′-CTCGAGCGGCCGCTCAATCATCGTAGTAATAATCTAACTTGGTG-3′. The product was digested with *Acc65I* and *NotI* and cloned into the *Acc65I* and *NotI* sites of pUAST.

The transgenic line #8 with UAS-htorA transgene on the second chromosome was used for the rescue experiments.

### HPLC analysis

Brains of third instar larvae or heads of 48–72 hr post-eclosion adult flies were homogenized in 0.1 M perchloric acid. Monoamines were separated by HPLC analysis using a CoulArray HPLC system (model 5600A; ESA, Chelmsford, MA) and a Synergi 4 µm Hydro-RP column (4.6×150 mm; Phenomenex, Torrance, CA) as described [21]. One hundred third instar larval brains or 75 to 200 adult heads were extracted in 100–200 µl of 0.1 M perchloric acid. Ten microliters of each extract were injected for each sample. Pool sizes were determined relative to freshly prepared standards (Sigma-Aldrich). Analysis was performed using ESA CoulArray software.

### Tyrosine hydroxylase assay

The specific activity of tyrosine hydroxylase (TH) in tissue extracts was determined as described previously [53] with some modifications. Briefly, 50 fly heads were homogenized in 100 µl of cold homogenization buffer. Homogenates were centrifuged at 9,300 x g for 10 minutes at 4°C. The protein concentration of the supernatants was determined using BioRad protein assay reagent (BioRad, Hercules, CA). Extracts were adjusted to equal protein concentrations (20–30 µg/µl), and 50 µl of the extract were mixed with 1 mM m-hydroxybenzylhydrazine dihydrochloride (NSD 1015; Sigma-Aldrich), 2 mM ferrous ammonium sulfate and 0.1% catalase. After incubation for 5 minutes at 37°C, 200 µM L-tyrosine and 1 mM (6R)-5,6,7,8-tetrahydro-L-biopterin (6R-BH4) were added to the mixture and incubated for another 20 minutes at 37°C. The reaction was terminated by adding 100 µl of stop solution (0.1 M perchloric acid, 0.4 mM sodium metabisulphite, and 0.1 mM EDTA) and the mixture was incubated on ice for 10 minutes. The assay mixture was centrifuged for 10 minutes at 1,000 x g at 4°C, and the supernatant was filtered through 0.2 µm PVDF microspin filters (Analytical, Pompton Plains, NJ) and analyzed by HPLC, as described above.

### GTP cyclohydrolase activity assay

Approximately 50 heads from 48–72 h post-eclosion adults were homogenized in 100 µl of cold 50 mM Tris, pH 8.0 solution containing 2.5 mM EDTA and 5% sucrose. After centrifugation of the homogenates at 9,300 x g for 10 minutes at 4°C, 5 mg of activated charcoal was added to 100 µl supernatant with gentle mixing to absorb eye pigment. Samples were centrifuged to remove the charcoal, and protein concentrations were determined using BioRad protein assay reagent. Forty-five µg of protein from tissue extracts was mixed with GTP to a final concentration of 0.25 mM in a final volume of 70 µl. After incubation at 37°C for 1 hour, the product of the reaction, 7,8-dihydroneopterin triphosphate, was oxidized to its fluorescent form in 30 µl of 1% iodine and 2% potassium iodide in 1 mM HCl, and incubated for 1 hour at room temperature. The samples were then centrifuged at 14,500 x g for 5 minutes at room temperature. The oxidation reaction was terminated and the samples decolorized with the addition of 15 l of 3% ascorbic acid. The samples were adjusted to pH 8.0, and 50 µl of each sample was mixed with 2 µl of calf intestine alkaline phosphatase, 7 µl of alkaline phosphatase buffer (Roche Applied Science, Indianapolis, IN) and 11 µl of distilled water. The mixtures were incubated at 37°C for 30 minutes to dephosphorylate the neopterin triphosphate product. The supernatant was filtered and then separated by HPLC as described above, except that neopterin was detected by fluorescence at excitation 360 nm/emission 465 nm, and its content was determined by comparison to commercial neopterin (Sigma-Aldrich) as standard using ESA CoulArray software.

### Western blot analysis

For analysis of whole flies, thirty adult male flies (*y w* or *y w dtorsin^KO13^*) were homogenized in 300 µl of RIPA buffer (50 mM Tris-HCl, pH 8.0, 150 mM NaCl, 1% NP40, 0.5% deoxycholate, 0.1% SDS (sodium dodecyl sulfate)), and the homogenate was sonicated and then clarified by a brief centrifugation (3,000 rpm for 2 min in a microcentrifuge). For detection of protein in adult heads, heads were removed manually from 50–60 flies of each genotype and were homogenized either in 100 µl phosphate-buffered saline (PBS) with Protein Inhibitor Cocktail (Roche Applied Science, Indianapolis, IN) or in 100 µl RIPA buffer, as above. The protein concentrations were determined with the Dc Protein Assay Reagent (Bio-Rad, Hercules, CA). The proteins (20–30 µg) were separated by electrophoresis in 10% SDS-polyacrylamide gels and transferred to nitrocellulose membranes. Membranes were blocked with 5% non-fat dry milk in PBS with 0.1% Tween 20 before incubation with primary antibodies. Rabbit anti-Dtorsin antibody raised against amino acids 51–339 of Dtorsin was a gift of Dr. Vijaya Ramesh (Massachusetts General Hospital, Boston, MA) and used at 1∶2000 dilution. GTPCH was detected using affinity-purified polyclonal anti-GTPCH isoform B/C antibody [54] and used at 1∶1000 dilution. TH was detected using rabbit anti-*Drosophila* TH [26] and was used at 1∶3000 dilution. Rabbit anti-Actin (Sigma-Aldrich) and mouse anti-Tubulin (Sigma-Aldrich) antibodies were used at 1∶200 and 1∶2000 dilutions. The secondary antibodies used were peroxidase-conjugated anti-rabbit IgG (GE Healthcare, Piscataway, NJ or Jackson ImmunoResearch, West Grove, PA) and peroxidase-conjugated anti-mouse (Abcam, Cambridge, MA). Signals were detected using Pierce ECL Western Blotting Substrate or Supersignal West Pico Chemiluminescent Substrate (Thermo Scientific, Rockford, IL).

### Larval locomotion assay and analysis

*y w dtorsin^KO13^* was balanced with *FM7i Act-GFP*. *dp cu Pu^Z22^ px sp* and *ple^2^* were balanced with the *CyO arm*-GFP and *TM3 Act*-GFP, respectively. GFP-negative first instar larvae were picked using a fluorescence microscope and transferred into the vials containing 1.5 g of FORMULA4-24 instant *Drosophila* media (Carolina Biological Supply Company, Burlington, NC) in 7 mls of water.

The larval locomotion assay was carried out at room temperature. Wandering third instar larvae of a particular genotype were picked from the vials. Male larvae were distinguished from females by the presence of larger genital discs as described previously [55]. A single larva was placed at the center of 0.7% agarose in a 100 mm petri dish plate for 30 seconds to recover from handling. The Petri dish was then placed on an evenly illuminated fluorescent light box (TW-26; VWR International, Radnor, PA).

Larval locomotion was recorded for 60 seconds using a Canon Powershot G7 attached to a Nikon SMZ800 microscope. Peristalsis frequency was counted manually from the QuickTime movie.

Larval movement was also recorded at 10 frames per second for 60 seconds using a digital video camera recorder (Handycam DCR-DVD108, Sony). Video tracking was performed using Prairie Dog, an application to trace the movement of an object in a series of TIFF files. Prairie Dog is a new application specifically developed by us to analyze the larval movement and is available upon request from H. M. Some locomotion analysis employed ICARUS software (Tracking/New User Feature) [56]. Larval head and tail locations were extracted and analyzed.

### Drug feeding assay

First instar larvae of the wild type (*y w*) and *y w dtorsin^KO13^* were transferred individually to 1.0 g FORMULA4-24 instant *Drosophila* media in 5 ml water or 5 ml of 20 mM dopamine-hydrochloride (Sigma-Aldrich), 20 mM octopamine hydrochloride (Sigma-Aldrich), or 10 mM serotonin hydrochloride (Sigma-Aldrich). Larval locomotion assays were performed, as described above.

### Antibody staining of larval brains and confocal analysis

Antibody staining of larval brains was carried out as described [57]. Anti-dTH antibody [26] was a gift from Dr. Wendi Neckameyer (St. Louis University, St. Louis, MO) and was used at a 1∶5000 dilution. The images were acquired by a Zeiss confocal microscope (LSM5 Pascal) and processed with Photoshop CS4. Multiple z-section confocal images were projected onto a single plane with NIH Image-J software (version 1.40) by Max intensity method.

## Supporting Information

Figure S1 **Isolation of *dtorsin* loss-of-function flies by homologous recombination.** **A**. Ends-out knockout targeting scheme, illustrating how a white mini-gene was inserted into the *dtorsin* genomic region, resulting in a complete deletion of the *dtorsi*n open reading frame while leaving the surrounding region intact. Other genes in the *dtorsin* genomic region are indicated by open boxes. Direction and location of primers that were used to make the knockout construct are shown by arrows. Locations of probes 1 and 2 (black boxes) and *BamHI* sites (B) are indicated. **B**. Southern blot of *BamHI* digested genomic DNA, confirming proper integration of the targeting construct. Probe 1 detected 1.5 kb *BamHI* fragment in the *BamHI* digested genomic DNA of the wild type (*y w*/Y and *w*/Y) males and 5.7 kb *BanHI* fragment in *y w dtorsin^KO13^*/Y and *w dtorsin^KO78^*/Y males. Probe 2 detected 0.5 kb *BamHI* fragment in the *BamHI*-digested genomic DNA of the wild type (*y w*/Y and *w*/Y) males and 5.7 kb *BanHI* fragment in *y w dtorsin^KO13^*/Y and *w dtorsin^KO78^*/Y males.
(TIF)

Figure S2 **Dtorsin immunoreactivity is reduced in dtorsin hemizygous adult extracts.** **A**. Anti-Dtorsin antibody recognizes a 38 kDa protein band in the wild type (*y w*) adult whole body (lane 1) and adult head (lane 3) extracts that is absent in *dtorsin*-null (*y w dtorsin^KO13^*) whole body (lane 2) and head (lane 4) extracts. Thirty µg of proteins were loaded in each lane. **B**. Anti-Actin antibody recognize a 42-kDa protein (*Drosophila* Actin) that are present in a similar amount both in the wild type (*y w*) adult whole body (lane 1) and adult head (lane 3) extracts as well as in *dtorsin*-null whole body (lane 2) and head (lane 4) extracts. Thirty µg of proteins were loaded in each lane.
(TIF)

Figure S3 **dtorsin loss-of-function mutant flies exhibit pigmentation and bristle phenotypes.** **A–C**, Pigmentation and bristle phenotypes of *dtorsin* mutants. Adult males of the genotype *y w*/Y (**A**), *y w dtorsin^KO13^*/Y (**B**), *y w dtorsin^KO13^*/Y; GDT101-2 (**C**). *dtorsin* null male *dtorsin^KO13^* (**B**) had shorter, thinner bristles with reduced pigmentation in the thorax compared to wild type (**A**). Both larger bristles (macrochaetae) and smaller bristles (microchaetae) were affected. These bristle phenotypes were completely rescued by the introduction of 1.9 kb genomic fragment containing the entire *dtorsin* gene (GDT101) (**C**).
(TIF)

Figure S4 **Effect of dopamine feeding on the adult pigmentation.** **A–D**, Effect of dopamine on the pigmentation phenotype of *dtorsin* mutants. Adult males of the genotype *y w* (**A**), *y w*+50 mM dopamine (**B**), *y w dtorsin^KO13^* (**C**), and *y w dtorsin^KO13^*+50 mM dopamine (**D**) flies. The pictures for **A–D** were taken one day after eclosion to ensure pigmentation reached the maximum level. Dopamine was added to the fly food during the larval period, as described in Materials and Methods.
(TIF)

Figure S5 **The double heterozygous combinations for dtorsin and DAT mutants have similar mobility compared to the wild type larvae.** Peristaltic frequencies for the wandering stage third instar larvae of the genotype *dtorsin^KO13^*/+; female (n = 7), and *dtorsin^KO13^*/+; *DAT^fumin^*/+ female (n = 14) are shown. Results are the mean ± S.E.M. No significant difference between the control females (*dtorsin^KO13^*/+) and the single heterozygous females (*dtorsin^KO13^*/+; *DAT^fumin^*/+).
(TIF)

Video S1 Shown is the crawling behavior of wild type (*y w*) third instar larva for 60 seconds. Wild type larvae showed smooth peristaltic movement of 40 to 60 strides per minute.
(MP4)

Video S2 Shown is the crawling behavior of *dtorsin* mutant male (*y w dtorsin^KO13^*/Y) third instar larva. *dtorsin* mutant larvae stopped more frequently and changed directions of movement more often than the wild type.
(MP4)

Table S1 (DOCX)
