# Correction: *dtorsin*, the *Drosophila* Ortholog of the Early-Onset Dystonia *TOR1A* (*DYT1*), Plays a Novel Role in Dopamine Metabolism

**DOI:** 10.1371/annotation/b76ab6b8-32c7-4f47-9ec9-9f302c9507f9

**Published:** 2011-10-31

**Authors:** Noriko Wakabayashi-Ito, Olugbenga M. Doherty, Hideaki Moriyama, Xandra O. Breakefield, James F. Gusella, Janis M. O'Donnell, Naoto Ito

The authors wish to add the following funding source to their financial disclosure information: Bachmann Strauss Dystonia-Parkinson Foundation (NI and XOB) (http://www.dystonia-parkinsons.org/) [^] 

