# Supplementary figures and images for: dtorsin, the Drosophila Ortholog of the Early-Onset Dystonia TOR1A (DYT1), Plays a Novel Role in Dopamine Metabolism
Source: PLoS One. 2011 Oct 12;6(10):e26183. doi: 10.1371/journal.pone.0026183 (PMC3192163; doi:10.1371/journal.pone.0026183)

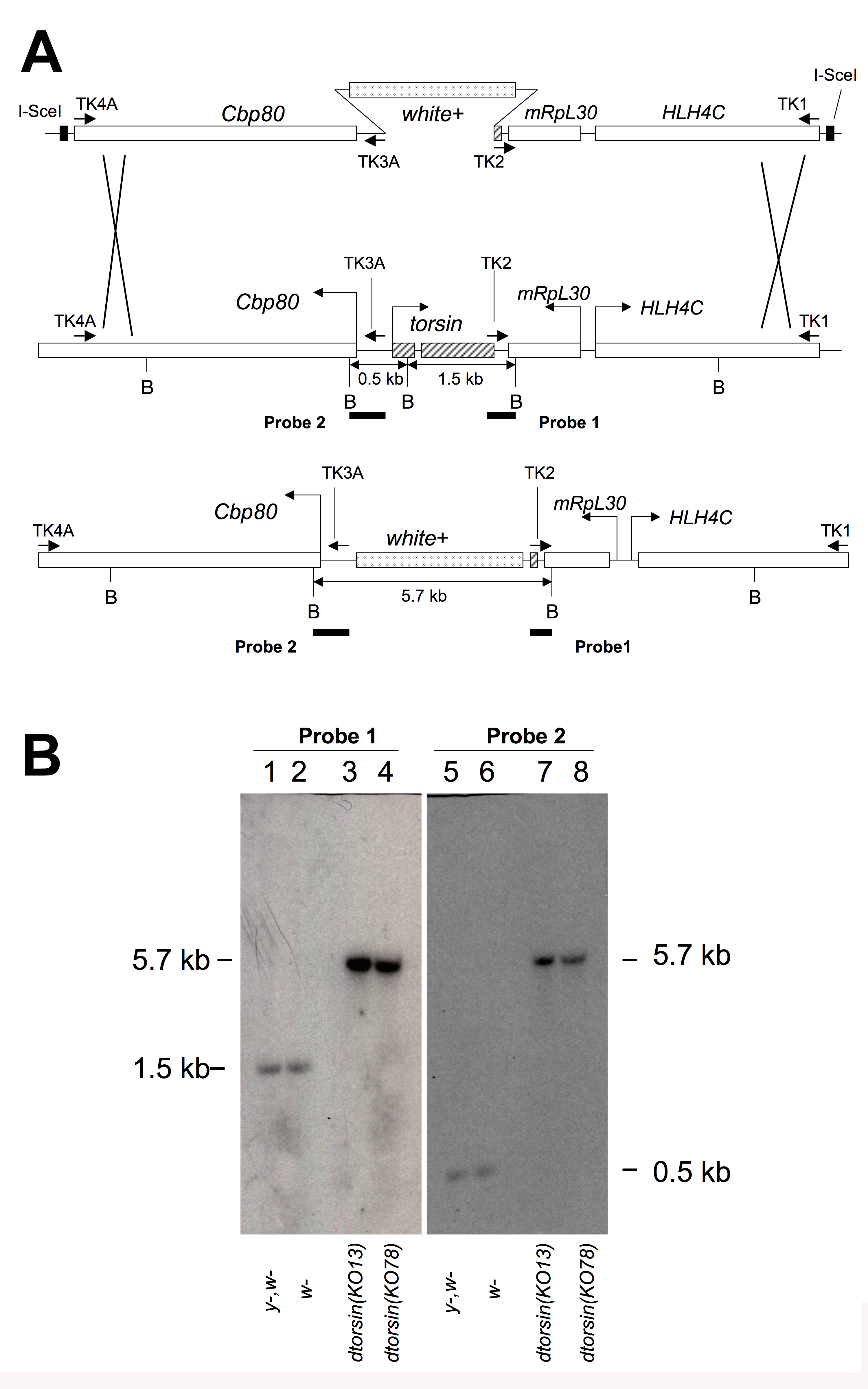

Supplement: Figure S1 — Isolation of dtorsin loss-of-function flies by homologous recombination. A. Ends-out knockout targeting scheme, illustrating how a white mini-gene was inserted into the dtorsin genomic region, resulting in a complete deletion of the dtorsin open reading frame while leaving the surrounding region intact. Other genes in the dtorsin genomic region are indicated by open boxes. Direction and location of primers that were used to make the knockout construct are shown by arrows. Locations of probes 1 and 2 (black boxes) and BamHI sites (B) are indicated. B. Southern blot of BamHI digested genomic DNA, confirming proper integration of the targeting construct. Probe 1 detected 1.5 kb BamHI fragment in the BamHI digested genomic DNA of the wild type (y w/Y and w/Y) males and 5.7 kb BanHI fragment in y w dtorsinKO13/Y and w dtorsinKO78/Y males. Probe 2 detected 0.5 kb BamHI fragment in the BamHI-digested genomic DNA of the wild type (y w/Y and w/Y) males and 5.7 kb BanHI fragment in y w dtorsinKO13/Y and w dtorsinKO78/Y males. (TIF) [file pone.0026183.s001.tif]

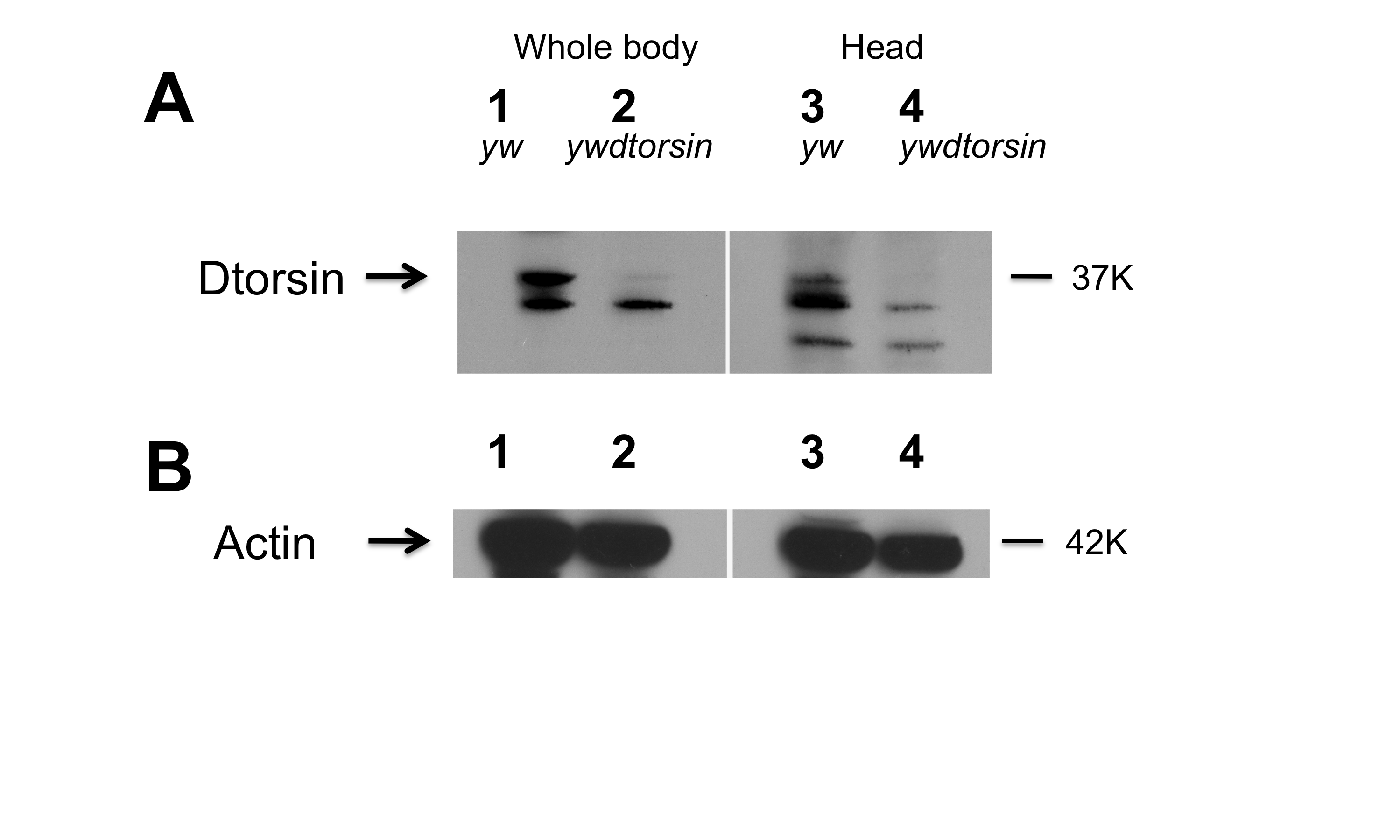

Supplement: Figure S2 — Dtorsin immunoreactivity is reduced in dtorsin hemizygous adult extracts. A. Anti-Dtorsin antibody recognizes a 38 kDa protein band in the wild type (y w) adult whole body (lane 1) and adult head (lane 3) extracts that is absent in dtorsin-null (y w dtorsinKO13) whole body (lane 2) and head (lane 4) extracts. Thirty µg of proteins were loaded in each lane. B. Anti-Actin antibody recognize a 42-kDa protein (Drosophila Actin) that are present in a similar amount both in the wild type (y w) adult whole body (lane 1) and adult head (lane 3) extracts as well as in dtorsin-null whole body (lane 2) and head (lane 4) extracts. Thirty µg of proteins were loaded in each lane. (TIF) [file pone.0026183.s002.tif]

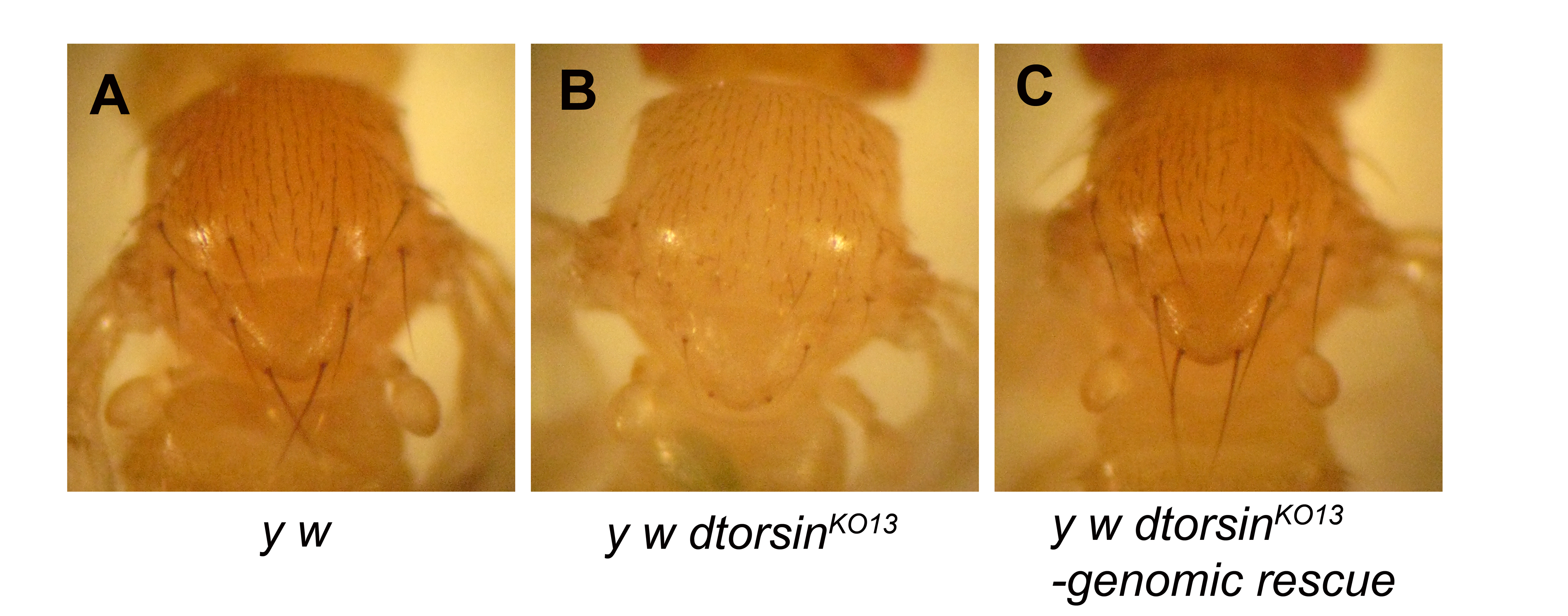

Supplement: Figure S3 — dtorsin loss-of-function mutant flies exhibit pigmentation and bristle phenotypes. A–C, Pigmentation and bristle phenotypes of dtorsin mutants. Adult males of the genotype y w/Y (A), y w dtorsinKO13/Y (B), y w dtorsinKO13/Y; GDT101-2 (C). dtorsin null male dtorsinKO13 (B) had shorter, thinner bristles with reduced pigmentation in the thorax compared to wild type (A). Both larger bristles (macrochaetae) and smaller bristles (microchaetae) were affected. These bristle phenotypes were completely rescued by the introduction of 1.9 kb genomic fragment containing the entire dtorsin gene (GDT101) (C). (TIF) [file pone.0026183.s003.tif]

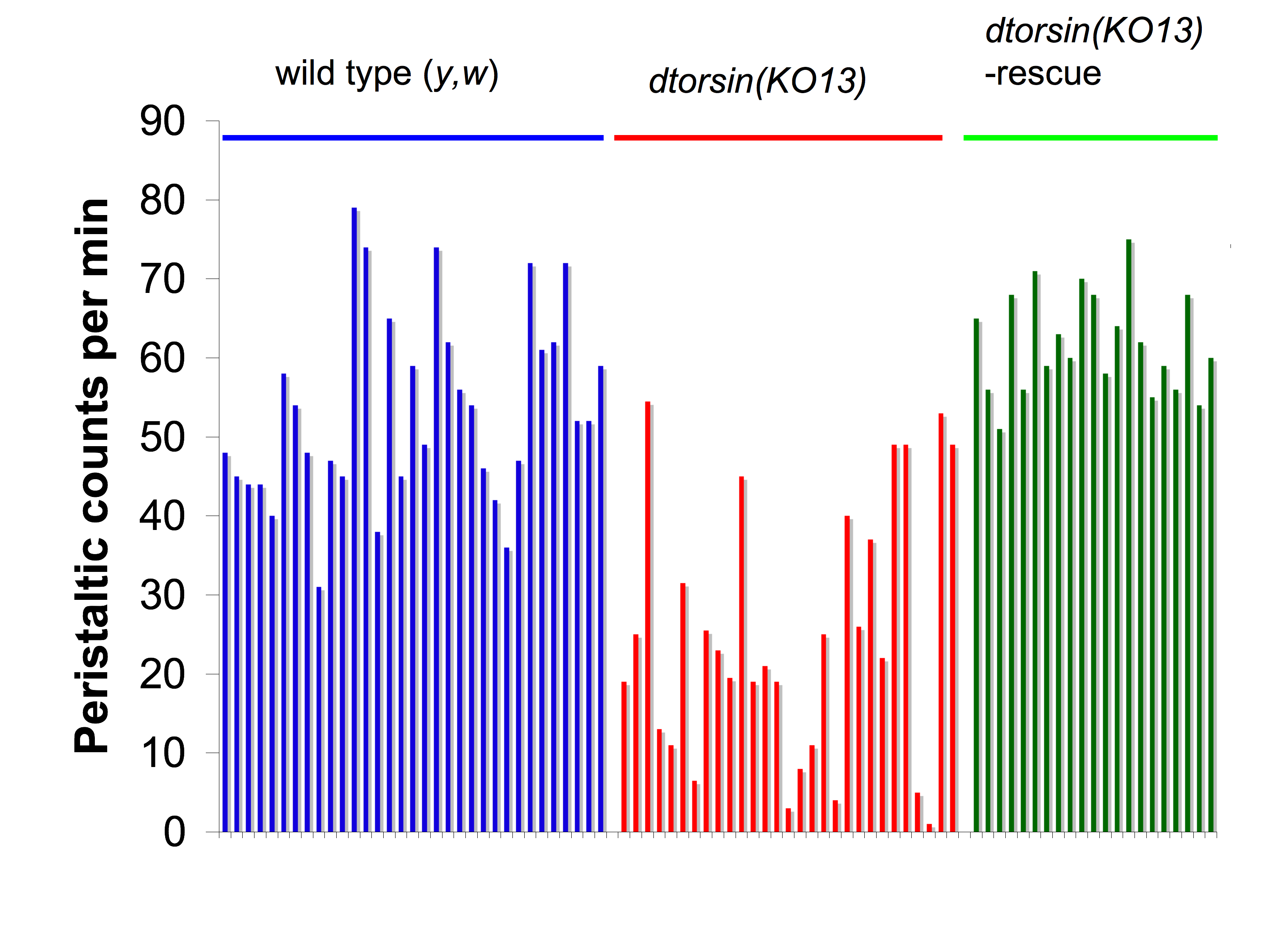

Supplement: Figure S4 — Effect of dopamine feeding on the adult pigmentation. A–D, Effect of dopamine on the pigmentation phenotype of dtorsin mutants. Adult males of the genotype y w (A), y w+50 mM dopamine (B), y w dtorsinKO13 (C), and y w dtorsinKO13+50 mM dopamine (D) flies. The pictures for A–D were taken one day after eclosion to ensure pigmentation reached the maximum level. Dopamine was added to the fly food during the larval period, as described in Materials and Methods. (TIF) [file pone.0026183.s004.tif]

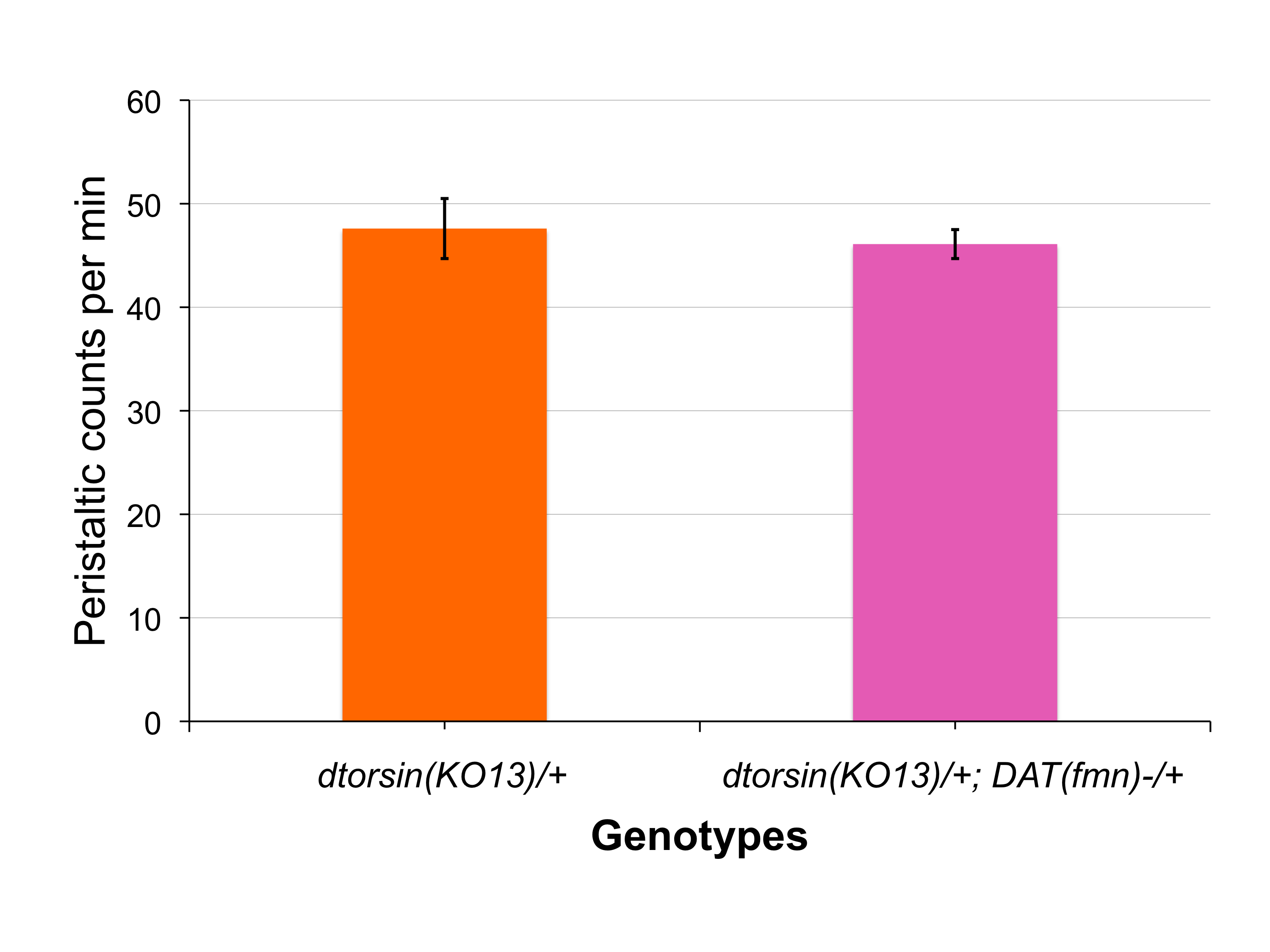

Supplement: Figure S5 — The double heterozygous combinations for dtorsin and DAT mutants have similar mobility compared to the wild type larvae. Peristaltic frequencies for the wandering stage third instar larvae of the genotype dtorsinKO13/+; female (n = 7), and dtorsinKO13/+; DATfumin/+ female (n = 14) are shown. Results are the mean ± S.E.M. No significant difference between the control females (dtorsinKO13/+) and the single heterozygous females (dtorsinKO13/+; DATfumin/+). (TIF) [file pone.0026183.s005.tif]
