# Supplementary material for: dtorsin, the Drosophila Ortholog of the Early-Onset Dystonia TOR1A (DYT1), Plays a Novel Role in Dopamine Metabolism
Source: PLoS One. 2011 Oct 12;6(10):e26183. doi: 10.1371/journal.pone.0026183 (PMC3192163; doi:10.1371/journal.pone.0026183)
Supplement: Table S1 — (DOCX) [file pone.0026183.s008.docx]

**Table S1. Rescue of *dtorsin* by the genomic fragment GDT101 and cDNA expression by GAL4 drivers.**

| Transgene (T) | *dtorsin^KO13^*/Y; T  males | *dtorsin^KO13^*/Y; T  males (%) | Total adult flies |
| --- | --- | --- | --- |
| Control (*y w*) | 0 | 0.0 | 146 |
| Genomic rescue  (GDT101-1) | 31 | 23.0 | 135 |
| Genomic rescue  (GDT101-2) | 47 | 35.1 | 134 |
| Act-GAL4/UAS-dtorsin | 22 | 16.1 | 137 |
| Tub-GAL4/UAS-dtorsin | 24 | 11.4 | 210 |
| Elav-GAL4/UAS-dtorsin | 62 | 17.1 | 363 |
| TH-GAL4/UAS-dtorsin | 0 | 0.0 | 302 |
| Act-GAL4/UAS-htorsinA | 1 | 0.34 | 291 |
| Tub-GAL4/UAS-htorsinA | 4 | 1.01 | 395 |

*dtorsin^KO13^/FM7c* heterozygote females were crossed with wild type control ( *y w* ) males or males with different transgenes (T) described on the left. Total number of adult flies and *dtorsin^KO13^*/Y; T males were counted. Percentage of *dtorsin^KO13^*/Y; T males among total adult flies was presented in the second column. GDT101-1 and GDT101-2 are independent transgenic lines carrying the 1.9 kb genomic rescue fragment (GDT101 transgene). Act-GAL4 and Tub-GAL4 are ubiquitously expressed GAL4 drivers. Elav-GAL4 is a pan-neuronal GAL4 driver. TH-GAL4 expresses GAL4 in dopamine producing cells.
